# Classifying *Cannabis sativa* chemovars using K-means analysis of elemental composition

**DOI:** 10.1186/s42238-025-00299-3

**Published:** 2025-11-24

**Authors:** Brendan Lukomski

**Affiliations:** Cambium Analytica, LLC 102 W Front Street, Traverse City, MI 49684 USA

**Keywords:** Chemometrics, Elemental Analysis, PCA, K-means, Cannabis

## Abstract

Understanding specific nutritional requirements of plants is a necessary component of growth optimization and can help growers promote desired traits and mitigate undesired traits. Cannabis is one such medicinally valuable plant prized for its content of specific phytochemicals. Cannabis is a commodity with numerous chemovars, each with their own nutritional needs. This can make generalizing targets for elemental content in the finished plant commodity difficult. One approach to make better informed decisions about cannabis plant nutrition is the application of chemometrics. In the current study, thirteen elements were quantitatively measured in the leaves of cannabis plants using an Agilent Inductively Coupled Plasma Mass Spectroscopy and an Elementar Vario Macro Cube. Correlation analysis, principal components analysis, and K-means clustering were utilized to describe and elucidate trends in the dataset. Moderately positive, monotonic correlations were found between magnesium, boron, and calcium, along with nitrogen, sulfur, and copper. PCA was used to corroborate these relationships. Clustering analysis was able to identify three distinct groups to which strains could be mapped with a relatively high degree of resolution when compared to cultivator identifiers. These findings suggest similar methods of introduction and elemental incorporation into the strains of these distinct groups. The method utilized in the current study demonstrate the ability of naïve clustering analysis to isolate differences in elemental concentrations between strains, allowing for the identification of unique cannabis chemovars. Such a process may be used to guide cultivation by classifying strains based on inherent nutritional requirements.

## Introduction

*Cannabis sativa* is a dioecious, short-photoperiod member of the Cannabaceae family that has long been cultivated for its pharmacological effects (Saloner 2023). These properties can be attributed to over 100 identified phytocannabinoids, a naturally occurring class of secondary metabolites, produced by the plant (Milan et al. 2024; Kalant and Kalant 2001). Of these cannabinoids, the non-psychoactive cannabidiol (CBD) and the psychoactive tetrahydrocannabinol (Δ^9^THC) have received the most attention due to their efficacy as treatments for seizures and chronic pain (Zafeiraki 2021; Bridgeman and Abazia 2017). Though these compounds are often cited to explain cannabis’ physiological effects, synthetically derived versions of these compounds have often displayed decreased efficacy compared to treatment with plant material itself (Bridgeman and Abazia 2017; Hazekamp et al. 2016). This suggests the presence of a physiological effect brought about by a combination of cannabis metabolites working in tandem, rather than any single compound producing a single effect (Hazekamp et al. 2016). This entourage effect, as it has been coined, emphasizes the importance of cultivating cannabis plants directly rather than synthetically deriving active compounds, meaning optimizing cultivation practices is of the utmost importance for producing a medicinally valuable product.

Cannabis is cultivated in two separate stages: vegetative and flowering (Saloner 2023; Chandra 2017). The vegetative phase primarily constitutes the period in which the plant develops its stems and leaves while the flowering phase marks its period of reproductive development (Saloner 2023). As a short-photoperiod plant, it will only begin reproductive development in response to an increase in time spent in darkness (Chandra 2017). In nature, this happens in response to decreased hours of sunlight in late summer and early fall, though in an indoor cultivation setting, this process can be manually triggered by regulating the plant’s exposure to light beneath grow lamps (Chandra 2017). Since the medicinally valuable phytocannabinoids are found in greatest abundance within the flower, influencing the development of the plant throughout its life cycle and into this vital period of growth is of primary concern to cultivators (Saloner 2023).

Various strains of cannabis have long been identified, differentiated from one another based on geographic region and structural differences (Hazekamp et al. 2016; Jin 2021). One such example differentiates between*sativa* and *indica*, the former of which was first cultivated in the Western world while the latter originated in South Asia (Hazekamp et al. 2016). However, due to extensive human cultivation and crossbreeding, these historical geographical identifiers of cannabis strain have since been called into question for use in the modern market (Chandra 2017; Jin 2021). Recent studies have reported moderate or nonexistent correlations between reported classification and both genetic and chemical profiles (Jin 2021; Herwig 2024). Updated classification schemes have sought to categorize strains by phytocannabinoid concentration, in which plants are labeled either drug-type or fiber-type based on their THC concentration (Saloner 2023; Hazekamp et al. 2016). However, in the recreational market, cannabis plants are cultivated for high THC content, and thus all belong to the same category, making this system of identification insufficient. Cultivators are aware of this and thus market their products using specific strain names that often have little basis in genetic or chemical composition (Hazekamp et al. 2016). Repeated studies have shown that these strain names rarely correlate with chemical composition, as several different strains can each possess the same underlying chemical profile (Hazekamp et al. 2016; Herwig 2024).

This ambiguity has led to a recent push in classifying cannabis based on chemical profile, grouping them into categories that are known as chemovars (Herwig 2024). Chemovars refer to specific strains of plants with a unique chemical makeup. These differences do not necessarily correlate with genetic predispositions to certain chemical profiles, as various environmental factors (Herwig 2024), cultivation conditions (Schober 2023; Danziger 2021), and locations within the plant (Danziger 2022)can have an impact on plant composition and thus produce distinct chemovars. Danziger et al was able to determine that variations in light exposure at different locations within the same plant result in a gradient of cannabinoid concentrations, with areas of greater light exposure possessing higher concentrations while areas of lower light exposure possess lower concentrations (Danziger 2021). Similarly, cannabinoid concentrations decreased as plant cultivation density increased, emphasizing the effect of cultivation practices on secondary metabolite production (Danziger 2022). Due to chemovar classification’s reliance and focus on chemical composition over genetics, it is the best predictor of physiological response for the consumer and has become the leading method of cannabis differentiation (Herwig 2024).

Cannabis chemovar classification is often focused on the active compounds within the plants: phytocannabinoids and terpenes (Jin 2021; Herwig 2024). Herwig et al were able to categorize cannabis strains based on terpene profiles, improving upon the*sativa/indica*grouping scheme that was common in the market (Herwig 2024). Hazekamp et al was likewise able to differentiate cannabis types using both terpene and cannabinoid profiles (Hazekamp et al. 2016). In both cases, chemical composition was proven to be a superior and more robust method for categorizing cannabis strains as opposed to the historical cultivar identifiers. While much of the work in classifying cannabis by chemovar has focused on cannabinoid and terpene concentrations, other chemical profiles within the plant hold the potential to do the same.

One such way cultivators influence this process is by supplementing plants with mineral nutrition (Saloner 2023; Shiponi 2021; Song et al. 2023). Since a plant’s chemical profile can be heavily influenced by environmental factors, cultivation practices play a large role in influencing chemovar assignment (Hazekamp et al. 2016; Herwig 2024). Depending on the intended final use of the plants, cultivation can be honed towards producing plants with certain characteristics, such as an increased concentration of a specific phytocannabinoid, through selective environmental conditions and nutritional supplementation (Saloner 2023). Mineral elements play a key role in influencing plant health and guiding physiological processes towards these specific ends (Saloner 2023). While cannabinoids are not produced in abundance during the vegetative stage, nutrition during this phase remains crucial in preparing the plant for high reproductive inflorescent yield and optimal cannabinoid production during the flowering phase (Kpai 2024; Shiponi and Bernstein 2021). During this stage, studies have demonstrated that low concentrations of magnesium reduce biomass accumulation, an important finding considering studies have also associated elevated levels of phosphorus and potassium with decreased magnesium concentration during this crucial developmental period (Shiponi 2021; Kpai 2024; Morad 2023). Likewise, Song et all was able to demonstrate that nitrogen deficiency throughout the vegetative phase resulted in an increase in cannabinoid concentration in the flowering phase due to the plant prioritizing carbon containing metabolites over nitrogen containing metabolites (Song et al. 2023). Due to the impacts experienced with both low concentrations and high concentrations of key nutrients, in addition to the impact and relationship with one another, mineral nutrition much be precisely honed to prevent undesirable outcomes (Kpai 2024; Shiponi and Bernstein 2021). The different effects resulting from variations in plant nutrition also imply the existence of optimal ranges for each of these mineral elements, likely influenced by both environmental conditions and inherent chemovar physiology (Saloner 2023; Shiponi and Bernstein 2021). Previous studies have begun to pinpoint these ranges, demonstrating that they are often genotype specific (Morad 2023). Since elemental dependencies change throughout the vegetative and flowering phases, understanding the plant’s current point in development and the unique nutritional needs at that point is critical to positively influencing growth and stimulating phytocannabinoid production (Saloner 2023; Kpai 2024; Shiponi and Bernstein 2021).

This study aimed to analyze the relationships among element concentrations in cannabis leaf tissue in the interest of identifying distinct elemental chemovars. Chemovars are characterized by their unique chemical makeup in comparison to other plants, meaning distinct chemovars will also possess distinct nutritional and cultivation requirements. The process outlined in this study will provide a model for naively classifying cannabis strains based on elemental concentrations, allowing the optimal mineral ranges of distinct chemovars to be identified and applied during the growth process. This study also provided greater insight into the standard mineral concentrations within the cannabis plant and their relationship to one another. Among similar works, no study could be found that possessed a dataset of this size. It is this study’s aim that, in outlining a methodology for cannabis, it may be applied to other medicinally valuable plants toward the end of optimizing cultivation and classifying chemovars.

## Methods

One thousand three hundred seventeen samples of cannabis leaf tissue were collected from plants at various points in the flowering phase of growth. The cannabis samples were donated by cannabis producers throughout Michigan and tested by Cambium Analytica, an independently contracted laboratory certified by the Michigan Cannabis Regulatory Agency (CRA) to conduct cannabis compliance testing. The sample space contains leaf tissue from throughout the flowering phase of growth and for a wide variety of chemovars grown under varying conditions, enabling broad generalization.

Due to the process by which tissue was collected, little information is available regarding specific cultivation practices. However, each tissue sample was provided with a producer specific strain name, allowing sample categorization based on marketed identifiers. While these strain names are useful in marketing, repeated studies have shown that they rarely correlate with chemovars, as a single chemovar can be sold under any number of different strain names (Hazekamp et al. 2016). This ambiguity serves as a driving motivator of this study, as classifying plants based on chemical composition removes the need for relying on inaccurate producer identifiers.

### Data collection

Tissue samples were prepared for elemental analysis in two separate ways. The first method involved digestion of an aliquot of tissue sample using a Milestone Ethos UP Microwave Digestion System. The resulting liquid was brought to volume in a 50 mL tube before being analyzed by an Agilent 7800 Inductively Coupled Plasma Mass Spectrometer (ICP-MS) affixed with an SPS 4 Autosampler. This instrument was utilized to measure concentrations of phosphorus (P), potassium (K), boron (B), zinc (Zn), nickel (Ni), molybdenum (Mo), manganese (Mn), magnesium (Mg), iron (Fe), copper (Cu), and calcium (Ca).

The second method involved the combustion of the sample using an Elementar Vario Macro Cube. This instrument was utilized to measure concentrations of both nitrogen (N) and sulfur (S). Together, these two analytical methods provided results for the complete list of thirteen nutritional analytes of interest.

Elemental analysis has long been utilized to guide plant cultivation, as understanding the functional response of the plant to mineral nutrition is essential to positively influencing growth (Menezes 2022). Cannabis, specifically, is a natural accumulator of macro and trace elements, and though the introduction of these elements is environmentally dependent, their retention has been suggested to be genetically determined (Zafeiraki 2021; Coffman 1975; Effect 2019). Understanding this distinction is important, as mineral nutrition has been linked to the production of several secondary metabolites, including the medicinally valuable cannabinoids (Saloner 2021). A lack of necessary nutrition, a form of abiotic stress on the plant, has also been demonstrated to increase secondary metabolism (Saloner 2022; Sampaio 2016).

### Statistical analysis

The Shapiro-Wilk test provided evidence to reject the normality assumption. Because of this, nonparametric methods were utilized in later analysis. The Kruskal-Wallis test was used to test for differences in the medians between groups (Emerson 2022; Chan et al. 1997). Following this, Dunn’s test was employed with a Bonferroni correction to adjust for repeated hypothesis tests (Dinno 2015). Spearman’s correlations were also calculated between pairwise groups of elements (Hauke 2011).

Principal components analysis (PCA) was also performed to reduce the complexity of the multivariate dataset and determine the primary sources of variance (Arumugam et al. 2012; Greenacre 2022; Peris-Diaz 2020). Utilizing the primary components, the K-means clustering algorithm was employed to examine groups given the unique strain identifiers (Kara 2009; Ikotun 2022). This was done in the interest of identifying whether the samples could be grouped appropriately and if elemental composition plays an underlying role in each strain.

## Results

To better understand the specifics of the elemental distributions within the leaf samples, descriptive statistics were calculated on the measured analyte concentrations. These values are given in Table 1.

**Table 1:** Descriptive statistics for each analyte

	Min	Median	Mean	Max	SD	RSD (%)
N	12800.00	47200.00	44971.75	75400.00	12889.69	28.66
Ca	6163.34	41804.06	44425.95	117215.36	15897.07	35.78
K	9767.47	26697.73	26732.15	48175.00	6316.85	23.63
Mg	2014.16	7703.00	8028.99	23306.93	2716.79	33.84
P	2372.64	5815.02	5911.25	13201.90	1485.39	25.13
S	0.00	3390.00	3421.55	23830.00	1143.53	33.42
Mn	15.11	147.80	177.52	830.50	124.15	69.93
Fe	33.99	106.58	142.33	1737.38	136.84	96.14
B	19.41	79.45	94.21	405.12	58.85	62.47
Zn	10.34	41.88	47.90	239.16	25.07	52.34
Cu	0.92	4.12	4.57	32.73	2.32	50.67
Mo	0.00	0.60	1.84	26.66	2.84	154.69
Ni	0.00	0.06	0.08	1.37	0.08	104.93

Values are given in ug/g (ppm) unless otherwise specified in the header row. Analytes are ordered by the average value in descending order

Measured concentrations range from a non-detectable minimum in the case of molybdenum and nickel to a maximum of 11.7% in the case of calcium. Of the elements measured, nitrogen, calcium, and potassium were the only three with averages greater than 1%, indicating these three elements are in greatest abundance within the plant. In contrast, manganese, iron, boron, zinc, copper, molybdenum, and nickel display average concentrations less than 0.02%, indicating these elements are consistently found in only trace amounts. The remaining elements: magnesium, phosphorus, and sulfur, fall between these groups of macro and micronutrients.

Use of the Shapiro-Wilk test provided evidence to reject the normality assumption for each of the analytes measured. The descriptive statistics in Table 1suggest most of these analytes are skewed right, as the concentration values possess lower bounds at a concentration of zero. Due to this normality violation, nonparametric tests were used in place of traditional parametric analysis (Feng 2014).

The Kruskal-Wallis test confirmed the presence of significant differences in the medians between groups (Emerson 2022; Chan et al. 1997). Dunn’s test then yielded insignificant*p*-values (values exceeding the significance threshold of 0.05) for the pairwise relationship of both nitrogen and calcium and manganese and iron (Dinno 2015). These results suggest their distributions are similar. Of these, nitrogen and calcium are the two most abundant elements in the samples tested.

### Correlations among trace elements

Nonparametric Spearman’s correlations were computed between pairwise groups of elements. These correlation coefficients are expressed using a correlation matrix, displayed in Table 2. The four coefficients with the greatest magnitudes are bolded.

**Table 2:** Spearman correlation analysis for the elemental concentrations of cannabis leaves

	N	P	K	B	Zn	Ni	Mo	Mn	Mg	Fe	Cu	Ca	S
N	1												
P	0.29	1											
K	0.45	0.25	1										
B	−0.24	0.10	0.03	1									
Zn	0.21	0.38	0.12	0.07	1								
Ni	0.24	−0.06	0.23	−0.11	0.12	1							
Mo	0.29	0.33	0.10	0.22	0.12	0.01	1						
Mn	−0.14	0.11	−0.10	0.21	0.51	0.02	0.01	1					
Mg	−0.06	0.22	0.03	**0.66**	0.15	−0.02	0.26	0.21	1				
Fe	0.24	0.23	−0.05	−0.01	0.27	0.12	0.40	0.22	0.09	1			
Cu	**0.59**	0.24	0.38	0.02	0.28	0.34	0.40	0.09	0.02	0.28	1		
Ca	−0.13	0.09	−0.04	0.44	0.38	0.08	−0.06	0.47	**0.63**	0.05	−0.09	1	
S	**0.61**	0.36	0.36	0.08	0.13	0.18	0.52	−0.12	0.09	0.25	0.52	−0.12	1

Coefficients do not represent linear correlations, but monotonic associations within samples. Coefficients with the four greatest magnitudes are marked in bold numbers

Magnesium and boron are the most heavily correlated of all the pairwise combinations (*r* = 0.66). Magnesium and calcium, nitrogen and sulfur, and nitrogen and copper also possess similarly moderate, positive correlations. Few pairs of elements displayed negative correlations. Of these, nitrogen and boron possessed a negative correlation with the greatest magnitude (*r* = −0.24).

### Principal components analysis

**Fig. 1:**
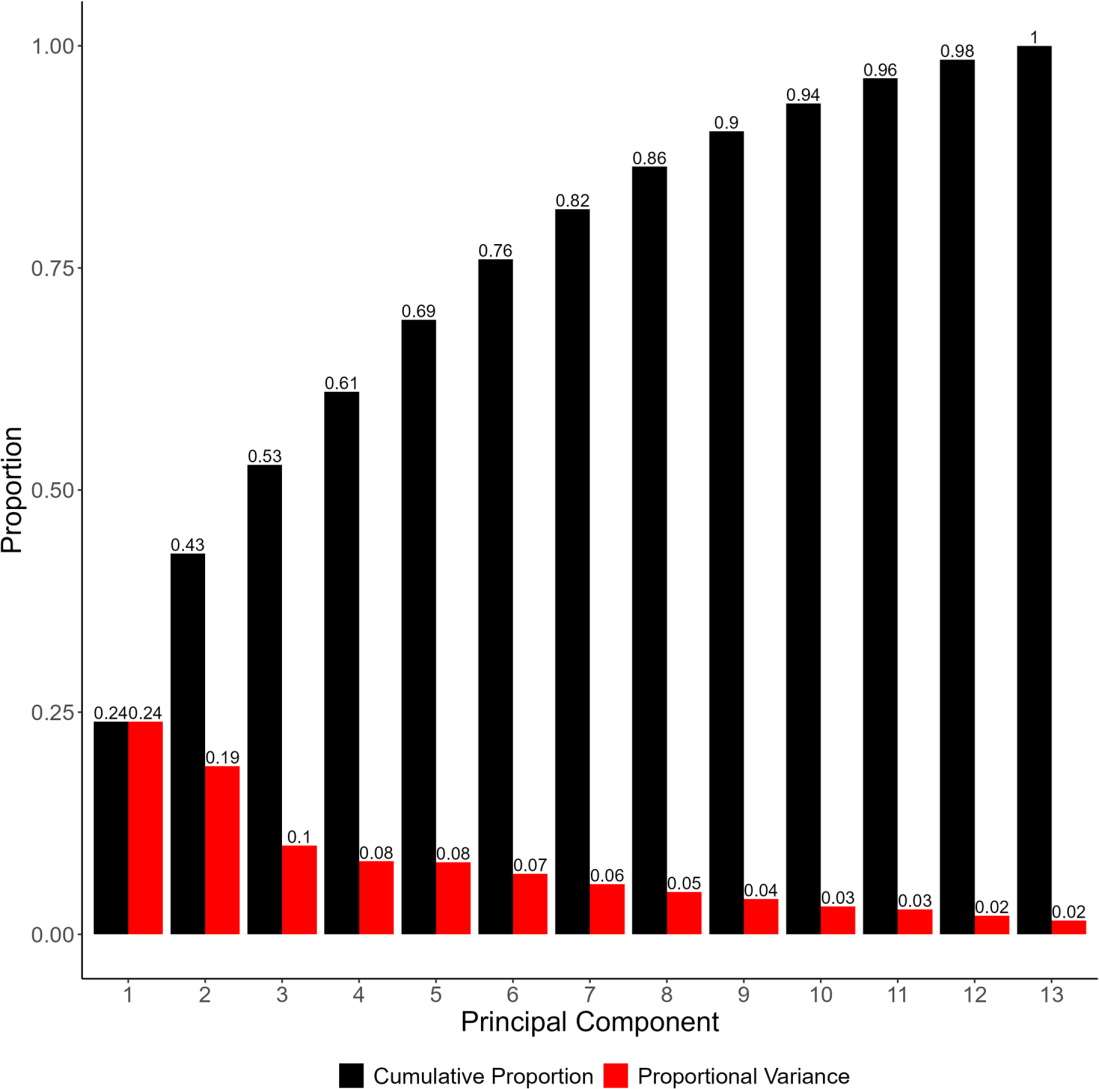
Proportional variance (red) and cumulative proportion (black) of each principal component in the PCA model. The proportional variance represents the percentage of variation of the original dataset explained by the component

Principal components analysis (PCA) was used to reveal three distinct clusters of elemental variances within the dataset. Conducting PCA on the dataset resulted in thirteen principal components. The proportion of the variance explained by each of the components, including the cumulative proportion, is expressed by the scree plot in Fig. 1.

**Fig. 2:**
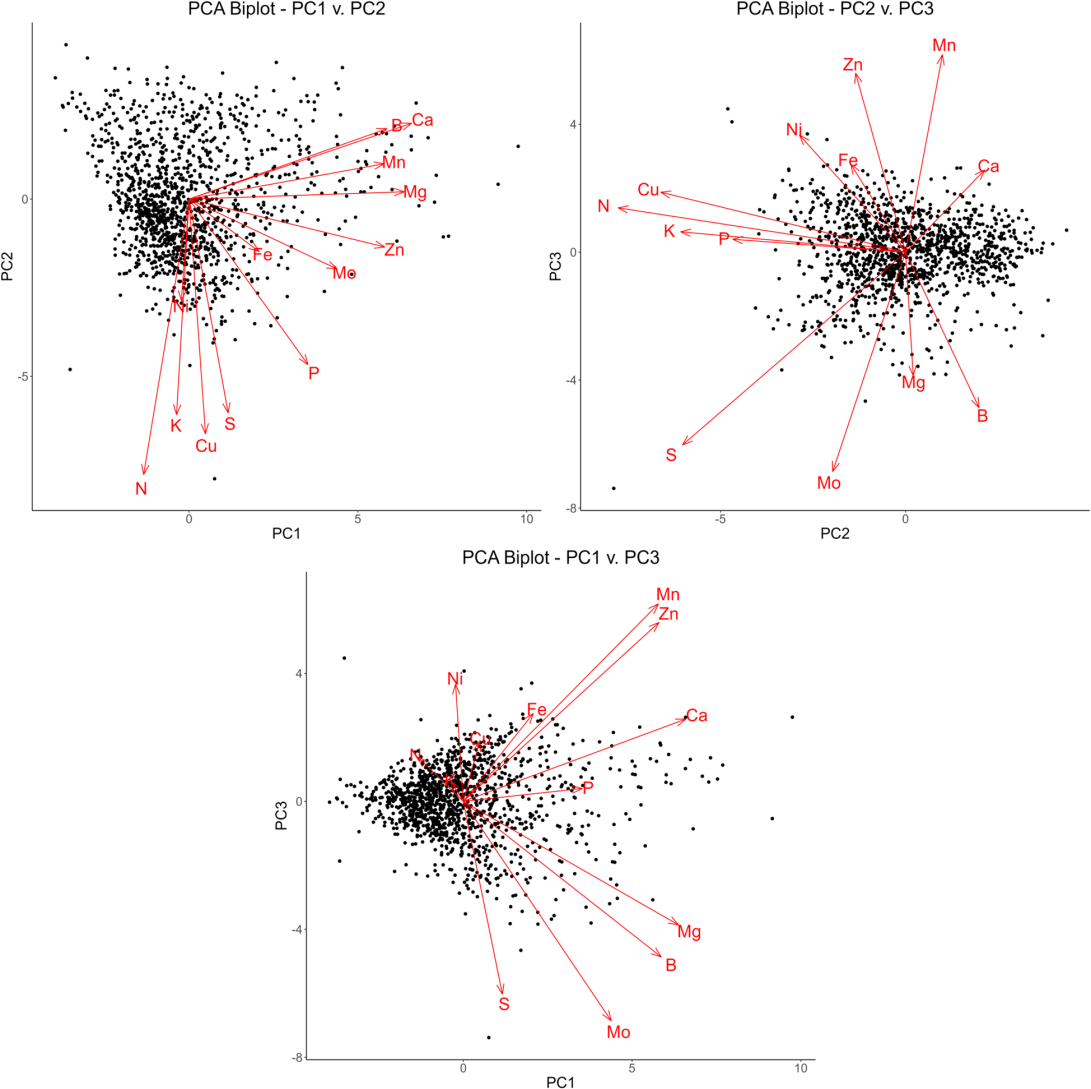
PCA biplots of PC1 v. PC2, PC2 v. PC3, and PC1 v. PC3. Individual variable loadings are expressed as red vectors, indicating the primary direction of variation for that analyte within each component

The first five principal components account for 69% of the total variation, yielding 24%, 18.7%, 10%, 8.2%, and 8.2% of the variance for each respectively. There is a noticeable drop after the second principal component, after which each subsequent component yields diminishing returns. Therefore, for the purpose of this analysis, only the first three principal components will be considered, as these account for over 50% of the variation. To better understand the specific relationships present within these principal components, biplots can be constructed utilizing the loadings and scores of each analyte (Taiz 2010; Greenacre 2022). These plots are expressed in Fig. 2.

In the plot of PC1 against PC2 in Fig. 2, the loading vectors for boron, calcium, manganese, magnesium, and zinc all have small angles in respect to one other while also being relatively parallel to the PC1 axis. These elements account for the most variation in both the first principal component and the dataset. They also present the highest correlations in Table 2, providing further evidence for this relationship. Likewise, the loading vectors for nitrogen, potassium, copper, and sulfur also possess small angles in relation to one another and are relatively parallel to the PC2 axis. Molybdenum, iron, and potassium fall in between these two groupings, appearing at an angle to both axes.

The second plot of PC2 against PC3 expresses the same relationship among nitrogen, potassium, copper, and sulfur, both against one other and the second principal component. The other analytes, however, do not appear to possess strong correlations among themselves or the third principal component. This indicates much of the strong correlation among these variables exists in the first two principal components.

Finally, the third plot of PC1 against PC3 shows much of the variation existing along the first principal component. This is to be expected, as the first principal component accounts for significantly greater variation than the third. Nickel, which exerts almost no influence on the first principal components, has a loading vector nearly parallel to the third principal component in a positive direction. This stands in contrast with the plot of PC1 against PC2, where nickel was parallel to the second principal component, but in a negative direction. This indicates variation in nickel is largely accounted for by the second and third principal component rather than the first.

This analysis confirms the presence of three specific groups of elements that tend to vary together, and in contrast to other elements. The specifics of these groupings, however, cannot be established by PCA. Other clustering algorithms are necessary to further elucidate these relationships.

### K-means corroborates the PCA clusters

The K-means clustering algorithm was utilized to identify three distinct clusters in the dataset. Three clusters were chosen as the optimal amount utilizing the silhouette method, modified to include scaled inertia (Rykov 2024). This agrees with the general clustering observed in the first two components of the PCA model.

**Fig. 3:**
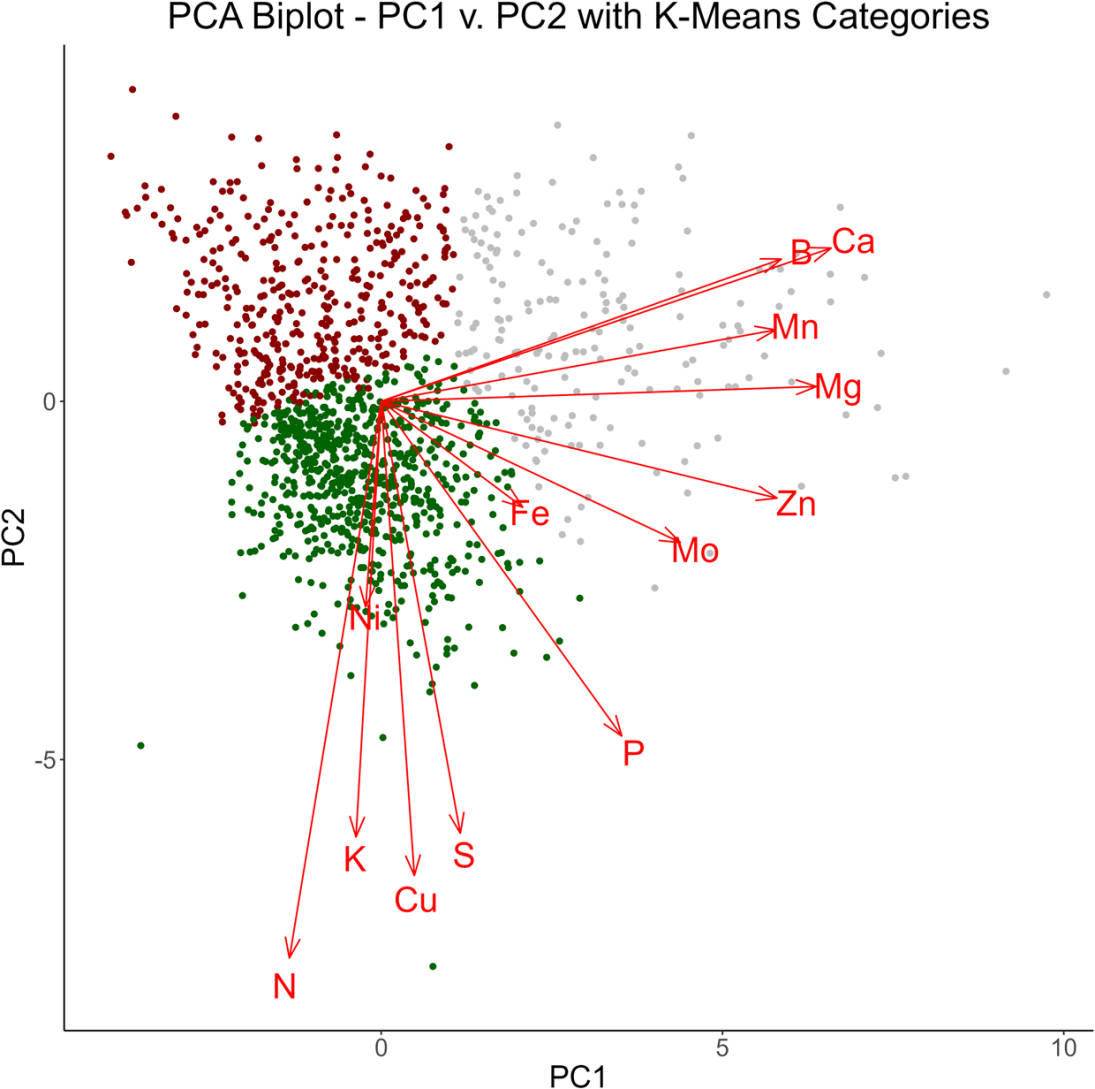
PCA biplot of PC1 v. PC2. Individual variable loadings are expressed as red vectors. Results of K-means clustering is denoted by colored points, with each of the three clusters expressed in a distinct color

Following K-means analysis, the clustering can be visualized by plotting against the first two principal components, as is expressed in Fig. 3.

**Fig. 4:**
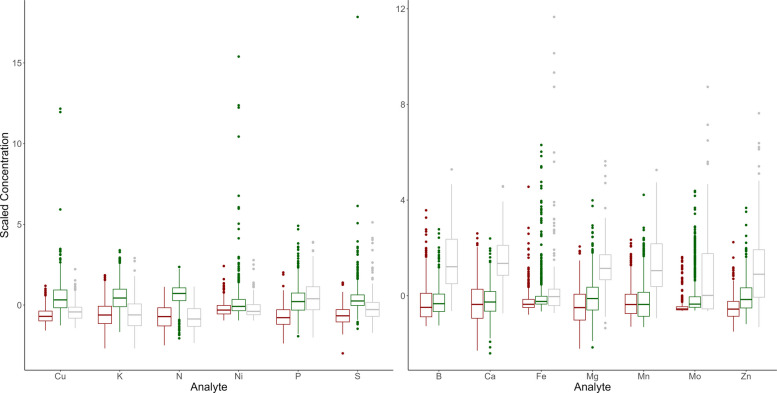
Distributions of key analytes within each K-means cluster. Group 2 has consistently higher values than group 1 in the first plot while group 3 has higher values than group 1 in the second plot

Overlaying this clustering over the biplot of the first two principal components can help interpret these clusters. The first grouping, denoted in red, lies in the same direction as the loading vectors for phosphorus, sulfur, copper, potassium, nickel, and nitrogen. This indicates the first cluster will tend to possess higher values for these elements. In contrast, the second grouping, denoted in green, lies in the opposite direction of these vectors, indicating lower values for these elements. This trend is further confirmed by examining the distributions of these scaled analytes in each of these clusters, which is expressed in the first plot of Fig. 4. The distribution of the red grouping tends to fall above the other two groups for each analyte present, while the distribution of the green grouping tends to fall below the other two.

Likewise, boron, calcium, manganese, magnesium, zinc, molybdenum, and iron are all directed toward the third cluster, denoted in gray. This indicates the third cluster will tend to possess higher values for these elements. The second plot in Fig. 4 corroborates this conclusion.

### Strain assignment to K-Means clusters

Utilizing the strain identifiers for each cultivator, frequencies of cluster assignment were determined for each distinct strain. Strains were discarded from analysis which possessed fewer than ten samples in order not to bias classifications with underrepresented strains. This resulted in 742 samples divided among twenty-three distinct strains. The percentage assignment of these samples to each of the three categories identified by the clustering algorithm are presented in Table 3. Dominant clusters are bolded.

**Table 3:** Strain specific groupings into K-means categories

Strain Code	Sample Count	Red	Green	Grey
C1-S4	10	**100**	0.00	0.00
C1-S2	15	**93.33**	0.00	6.67
C1-S10	63	**66.67**	31.75	1.59
C1-S8	45	**66.67**	17.78	15.56
C1-S12	50	**58.00**	36.00	6.00
C1-S1	28	**57.14**	39.29	3.57
C1-S3	106	**44.34**	34.91	20.75
C2-S4	23	8.70	**86.96**	4.35
C2-S10	25	8.00	**84.00**	8.00
C2-S8	31	16.13	**83.87**	0.00
C2-S23	15	20.00	**80.00**	0.00
C2-S7	19	21.05	**78.95**	0.00
C2-S2	36	16.67	**63.89**	19.44
C2-S9	37	35.14	**62.16**	2.70
C1-S11	20	30.00	**55.00**	15.00
C2-S3	32	34.38	**52.12**	12.50
C1-S7	77	31.17	**45.45**	23.38
C3-S3	11	0.00	27.27	**72.73**
C3-S1	14	7.14	28.57	**64.29**
C5-S1	27	3.70	33.33	**62.96**
C5-S3	18	5.56	38.89	**55.56**
C3-S2	13	0.00	46.15	**53.85**
C5-S2	27	7.41	40.74	**51.85**

Category colors denote the percent inclusion of each strain into the categories described in Fig. 4. Dominant clusters are bolded

It is evident that several strains were able to be grouped almost entirely into a single cluster. As evidenced by Fig. 4, strains C2-S4, C2-S10, and C2-S8 are almost entirely contained within the first cluster, meaning they tend to have higher concentrations of copper, potassium, nitrogen, nickel, phosphorus, and sulfur in comparison to the other two groups. Likewise, strains C1-S4 and C1-S2 are almost entirely contained within the second cluster, indicating they would tend to have lower concentrations of those same elements. Strains present in the third cluster would therefore tend to possess higher values for boron, calcium, iron, magnesium, manganese, molybdenum, and zinc.

The dominant strains within each cluster also appeared to have been sourced from the same producers. The cultivator identifier, denoted as the first half of each strain code, indicates cultivator 1’s plants are almost entirely dominant within the first cluster. Similarly, cultivator 2’s plants are all dominant within the second cluster. Both cultivator 3 and 5 are completely dominant within cluster 3. This finding suggests that the underlying cultivator of the plant may contribute to this elemental clustering.

## Discussion

The data collected in this study was used to characterize relationships that exist between trace elements in cannabis leaves which can be and were then used to classify strains by common elemental compositions via clustering statistics. The results suggest certain strains possess similar elemental nutrition compositions and can be categorized reliably into groups. These groupings may provide insigh into the response of plants to growing conditions. However, extrinsic environmental factors, such as mineral supplementation and abiotic exposure of elements to the plant, may introduce confounding factors for classifying chemovars between producers.

The distribution of elemental concentrations, in addition to several pairwise correlations, found in cannabis were similar to those expressed in other agricultural plants (Taiz 2010). Cations such as magnesium, potassium, and calcium have been shown to competitively inhibit one another during root uptake (Morad 2023). This seems to contradict the identification of the strong positive association experienced between magnesium and calcium that has also been attested to in other angiosperm species (White 2018). However, since this sample set consists of nutritional data from many separate strains, this positive association may be attributed to plants with a higher concentration of one cation having a higher concentration of other cations as well. This may be attributed to inherent physiology predisposing a specific strain to higher elemental concentrations or variations in cultivation practices to result in increased mineral uptake (Morad 2023; White 2018). Further study on this association may examine cation associations over time within the same plant, which would serve to demonstrate how each element responds to changes in concentration of others. To our knowledge, the remaining strong correlations among elements are not mentioned in established literature.

Three distinct groupings of analytes were identified using both PCA and the K-means clustering algorithm, possibly indicating similar modes of element incorporation among them. An underlying genetic factor may also be at work, as different strains may possess their own nutritional requirements expressed in varying trace metal concentrations.

When classified based on strain identifiers, these groupings become apparent, as several strains were able to be classified almost entirely into single clusters. For instance, strains C2-S4, C2-S10, and C2-S8 are almost entirely contained within the first cluster while strains C1-S4 and C1-S2 are almost entirely contained within the second. Other strains, such as C1-S7 and C1-S3, were evenly divided among all three clusters, indicating they likely contain characteristics of all three, suggesting the presence of a hybrid chemovar.

The groupings may also be explained by varying extrinsic cultivation practices, including soil composition and fertilizer regimens. The first half of each strain code contains the cultivator identifier, of which four were utilized in this analysis. Of these four, strains from cultivator two were entirely dominant in group one while strains from cultivator one was predominantly dominant in group two. Likewise, cultivators three and five were primarily dominant in group three. The clustering of each cultivators’ strains indicates similar elemental compositions within plants produced at each facility. Given the strong influence of environmental factors on chemical composition, this outcome is not surprising. Varying growth conditions between facilities, such as fertilizer regiments and soil conditions, could explain this relationship. This finding reveals a possible application of this methodology in identifying a plant’s cultivator based on elemental composition, as utilizing the K-means classification model revealed grouping are often dominated by a single producer.

Application of these models may provide a means to naively categorize cannabis strains by chemical data. In doing so, strains may be identified and classified based on chemovar, as was demonstrated in categorizing the various strains provided by different cultivators. These findings illustrate the value in chemometric analysis of cannabis, an application which has still received little attention since the widespread legalization of the plant for medicinal and recreational use. Mineral elements play key roles in a variety of biochemical pathways that support plant health and generate secondary metabolites, among which are the medicinally valuable cannabinoids. The application of the methodology outlined in this study, in conjunction with additional data relating to concentrations of these key analytes, could serve to aid cultivation and crop optimization of plant nutrition (Saloner 2021).

## Data Availability

I have attached the full dataset under the'Related Files'field for consideration by the editor. However, on the public side, the data that supports the findings of this study are available from Cambium Analytica, LLC but restrictions apply to the availability of these data, which were used under license for the current study, and so are not publicly available. Data are however available from the authors upon reasonable request and with permission of Cambium Analytica, LLC.

## Abbreviations

PCA: Principal Components Analysis
ICP-MS: Inductively Coupled Plasma Mass Spectrometry
CRA: Cannabis Regulatory Agency
CBD: Cannabidiol
THC: Tetrahydrocannabinol
